# Supplementary material for: Fat-to-Muscle Ratio Is Independently Associated with Hyperuricemia and a Reduced Estimated Glomerular Filtration Rate in Chinese Adults: The China National Health Survey
Source: Nutrients. 2022 Oct 8;14(19):4193. doi: 10.3390/nu14194193 (PMC9573307; doi:10.3390/nu14194193)
Supplement: Supplementary file 1 [file nutrients-14-04193-s001.zip › nutrients-1941360-supplementary.pdf]

Fat-to-Muscle Ratio Is Independently Associated with Hyperuricemia and a Reduced Estimated Glomerular Filtration Rate in Chinese Adults: The China National Health Survey

Supplementary materials

Table S1. The cross-over prevalence of hyperuricemia and reduced eGFR in FMR groups, stratified by sex

	Male				Female			
**Age-groups**	**HUA cases**	**Prevalence**	**Reduced eGFR cases**	**Prevalence**	**HUA cases**	**Prevalence**	**Reduced eGFR cases**	**Prevalence**
20–	371	28.89	164	12.77	156	7.79	132	6.59
30–	596	29.83	438	21.92	197	6.46	418	13.71
40–	798	25.21	1001	31.62	375	7.52	1300	26.07
50–	706	22.59	1161	37.15	646	13.94	1727	37.28
60–	446	20.73	1050	48.81	553	18.56	1524	51.16
70–80	170	21.28	508	63.58	229	22.95	670	67.13
Overall	3087	24.65	4322	34.51	2156	11.56	5771	30.95

HUA: hyperuricemia; eGFR: estimated glomerular filtration rate, mL/min per 1.73 m^2.

Table S2. The independent effect of FMR on hyperuricemia in the study population.

	Men								Women							
	Model 1				Model 2				Model 1				Model 2			
**Overall**	OR	95% CI		P	OR	95% CI		P	OR	95% CI		P	OR	95% CI		P
**FMR**																
Q2	1.686	1.384	2.055	<0.001	1.604	1.317	1.953	<0.001	1.920	1.572	2.346	<0.001	1.914	1.564	2.342	<0.001
Q3	2.639	2.217	3.141	<0.001	2.312	1.907	2.803	<0.001	2.819	2.373	3.350	<0.001	2.671	2.124	3.360	<0.001
Q4	3.693	3.147	4.334	<0.001	2.713	2.145	3.433	<0.001	5.621	4.685	6.744	<0.001	4.473	3.396	5.892	<0.001
**Age < 40**																
**FMR**																
Q2	1.876	1.415	2.488	<0.001	1.754	1.300	2.366	<0.001	2.219	1.445	3.408	<0.001	2.195	1.439	3.350	<0.001
Q3	3.369	2.601	4.365	<0.001	2.842	1.965	4.110	<0.001	3.087	2.294	4.156	<0.001	2.463	1.557	3.897	<0.001
Q4	4.304	3.186	5.813	<0.001	2.580	1.549	4.299	<0.001	11.031	8.022	15.168	<0.001	6.271	3.354	11.726	<0.001
**40≤ Age <60**																
**FMR**																
Q2	1.761	1.357	2.286	<0.001	1.617	1.230	2.127	0.001	2.013	1.492	2.715	<0.001	2.016	1.493	2.721	<0.001
Q3	2.737	2.084	3.594	<0.001	2.261	1.693	3.020	<0.001	3.221	2.389	4.342	<0.001	3.075	2.166	4.364	<0.001
Q4	3.696	2.974	4.593	<0.001	2.676	1.992	3.595	<0.001	5.243	3.930	6.994	<0.001	4.285	2.864	6.412	<0.001
**Age ≥60**																
**FMR**																
Q2	1.478	0.931	2.347	0.0952	1.486	0.940	2.348	0.088	1.979	1.289	3.037	0.003	1.984	1.293	3.044	<0.001
Q3	1.985	1.425	2.764	0.0002	2.019	1.286	3.171	0.003	2.472	1.692	3.611	<0.001	2.411	1.551	3.747	0.003
Q4	3.281	2.214	4.861	<.0001	3.002	1.773	5.082	<0.001	5.281	3.425	8.142	<0.001	4.549	2.623	7.889	<0.001

Model 1 was adjusted for age, rural/urban places, educational level, study sites, alcohol drinking status (male only), smoking status (male only), hypertension, diabetes, dyslipidemia, and serum creatine. Model 2 was additionally adjusted for body mass index based on Model 1. FMR: fat-to-muscle ratio; Q2–Q4: the numbers fall in the 25–49$_{th}$, 50–74$_{th}$ and 75–100$_{th}$ range of FMR. OR: odds ratio; CI: confidence interval.

Table S3. The independent effect of FMR on reduced estimated glomerular filtration rate in the male study population aged 20–59.

	**Model 1**				**Model 2**				**Model 3**			
**Overall**	OR	95% CI		P	OR	95% CI		P	OR	95% CI		P
**FMR**												
Q2	1.326	1.147	1.532	<0.001	1.328	1.082	1.631	0.008	1.261	1.029	1.546	0.027
Q3	1.350	1.167	1.563	<0.001	1.334	1.042	1.708	0.023	1.188	0.931	1.515	0.161
Q4	1.429	1.230	1.661	<0.001	1.410	1.038	1.916	0.029	1.224	0.917	1.632	0.165
**Age <40**												
**FMR**												
Q2	0.980	0.759	1.265	0.877	0.941	0.663	1.334	0.726	0.840	0.594	1.187	0.314
Q3	1.096	0.834	1.439	0.511	0.991	0.664	1.480	0.966	0.795	0.537	1.177	0.244
Q4	0.974	0.732	1.297	0.857	0.898	0.499	1.619	0.715	0.702	0.410	1.202	0.191
**40≤ Age <60**												
**FMR**												
Q2	1.549	1.297	1.849	**<0.001**	1.549	1.236	1.942	**<0.001**	1.483	1.179	1.866	**0.001**
Q3	1.528	1.281	1.822	**<0.001**	1.528	1.152	2.027	**0.004**	1.380	1.045	1.821	**0.024**
Q4	1.704	1.421	2.042	**<0.001**	1.669	1.177	2.369	**0.005**	1.454	1.037	2.040	**0.031**

Model 1 was adjusted for age, rural/urban places, educational level, alcohol drinking status, smoking status, hypertension, diabetes, and dyslipidemia. Model 2 was additionally adjusted for body mass index based on Model 1. Model 3 was additionally adjusted for hyperuricemia based on Model 2. FMR: fat-to-muscle ratio; Q2–Q4: the values fall in the 25–49th, 50–74th and 75–100th centile of FMR. OR: odds ratio; CI: confidence interval.

Table S4. The independent effect of FMR on reduced estimated glomerular filtration rate in the female study population aged 20–59.

	**Model 1**				**Model 2**				**Model 3**			
**Overall**	OR	95% CI		P	OR	95% CI		P	OR	95% CI		P
**FMR**												
Q2	1.067	0.962	1.183	0.215	1.058	0.958	1.168	0.259	1.023	0.930	1.126	0.633
Q3	1.162	1.052	1.284	0.004	1.152	1.049	1.264	0.004	1.067	0.930	1.224	0.348
Q4	0.954	0.837	1.088	0.474	0.947	0.832	1.077	0.395	0.818	0.663	1.008	0.059
**Age <40**												
**FMR**												
Q2	1.019	0.821	1.265	0.862	1.018	0.818	1.267	0.871	0.966	0.793	1.177	0.725
Q3	1.162	0.880	1.534	0.282	1.143	0.736	1.774	0.542	1.077	0.701	1.654	0.728
Q4	1.006	0.696	1.455	0.972	1.139	0.599	2.167	0.685	0.944	0.507	1.759	0.787
**40≤ Age <60**												
**FMR**												
Q2	1.056	0.941	1.185	0.342	1.057	0.941	1.186	0.342	1.026	0.917	1.148	0.650
Q3	1.137	1.023	1.264	0.019	1.127	0.957	1.328	0.148	1.062	0.900	1.252	0.468
Q4	0.932	0.808	1.077	0.332	0.885	0.705	1.109	0.280	0.798	0.634	1.005	0.055

Model 1 was adjusted for age, rural/urban places, educational level, hypertension, diabetes, and dyslipidemia. Model 2 was additionally adjusted for body mass index based on Model 1. Model 3 was additionally adjusted for hyperuricemia based on Model 2. FMR: fat-to-muscle ratio; Q2–Q4: the values fall in the 25–49th, 50–74th and 75–100th centile of FMR. OR: odds ratio; CI: confidence interval.

Table S5. The association between hyperuricemia and reduced estimated glomerular filtration rate in the study population.

	Male				Female			
	OR	95% CI		P	OR	95% CI		P
Overall	2.834	2.544	3.158	<0.001	2.957	2.590	3.376	<0.001
**FMR stratified**								
Q1	3.568	2.708	4.701	<0.001	3.726	2.545	5.457	<0.001
Q2	2.803	2.286	3.436	<0.001	2.923	2.037	4.195	<0.001
Q3	2.947	2.429	3.575	<0.001	2.962	2.180	4.024	<0.001
Q4	2.679	2.252	3.187	<0.001	2.855	2.473	3.295	<0.001
**Age-stratified**								
20–	2.472	1.963	3.113	<0.001	2.935	2.081	4.138	<0.001
40–	2.656	2.355	2.995	<0.001	3.020	2.634	3.462	<0.001
60–80	4.503	3.578	5.666	<0.001	3.040	2.457	3.760	<0.001
**Urban-rural stratified**								
Urban	2.681	2.338	3.074	<0.001	2.883	2.440	3.408	<0.001
Rural	3.294	2.641	4.108	<0.001	3.141	2.532	3.895	<0.001

The covariates adjusted for were age, body mass index, urban/rural place, education, smoking status (males only), alcohol drinking status, hypertension, dyslipidemia, and diabetes. OR: odds ratio; CI: confidence interval. FMR: fat-to-muscle ratio; Q1–Q4: the values fall in the 0–24th, 25–49$_{th}$, 50–74$_{th}$ and 75–100$_{th}$ centile of FMR.
